# How Mountain Park Spatial Environments Affect Physiological and Psychological Perceptions of Young Adults Based on Real Time Sensor Monitoring

**DOI:** 10.3390/s26134177

**Published:** 2026-07-02

**Authors:** Xinyu Yang, Changjuan Hu, Cong Gong

**Affiliations:** 1 Faculty of Architecture and Urban Planning, Chongqing University, Chongqing 400045, China; 202315021008@stu.cqu.edu.cn (X.Y.); changjuanhu@cqu.edu.cn (C.H.); 2 Key Laboratory of New Technology for Construction of Cities in Mountain Area, Ministry of Education, Chongqing University, Chongqing 400045, China

**Keywords:** mountainous urban parks, gathering spaces, young adults, visual and auditory perception, on-site experiment, random forest model

## Abstract

Gathering spaces within urban parks serve as primary outdoor leisure venues, playing a critical role in facilitating social interaction and restoring the physical and mental well-being of this demographic. This study uses the example of Pipa Mountain Park in Chongqing, China to explore the psychological and physiological perceptual effects of spatial environmental characteristics on young adults in four typical gathering spaces: path platform, elevated point, viewing boundary, and key node. To this end, we employed onsite experimental methods using wearable ergonomic devices to collect participants’ physiological data, including electrophysiological, electroencephalogram (EEG), and eye-tracking data. Visual and auditory psychological perception evaluation data were obtained through on-site questionnaires. Descriptive statistical analysis revealed differential trends in participants’ psychological perceptions and physiological responses across distinct gathering spaces. The elevated point demonstrated the most favorable ratings for the psychological dimension “comfort” (M = 1.63, SD = 2.09). Subsequent principal component analysis elucidated key psychological perception indicators in mountainous settings, while Friedman test, Kruskal–Wallis tests, and random forest modeling quantified the effects of specific spatial environmental indicators on perceptual responses. Results indicated significant differences in psychological perceptions and physiological responses across gathering space typologies (*p* < 0.05). Influenced by the preferences and behavioral habits of young adults, environmental element complexity significantly enhanced attentional engagement (χ^2^ = 68.428, *p* < 0.01) and facilitated positive perceptual responses. The synergistic effects of the visual and auditory elements significantly enhance the restorative benefits of space; however, poor accessibility weakens this advantage. This study provides evidence for the in-depth analysis of the intrinsic mechanisms between the spatial environment and multisensory perception in urban mountain parks.

## 1. Introduction

According to data from the World Health Organization (WHO), mental health disorders are among five of the top ten leading causes of disability worldwide [1]. In the post-pandemic era, the incidence of psychological crises such as depression, anxiety, stress, and fear has surged significantly among young adults, drawing widespread attention to their mental well-being [2,3]. A substantial body of research indicates that urban green spaces and parks provide essential opportunities for young adults to connect with nature, thereby facilitating social interactions and enhancing their mental health. As a primary component of green space systems in mountainous cities, mountainous urban parks offer rich auditory and visual resources and play a significant role in sustainable urban built environments [4,5,6]. Gathering spaces, which form in mountainous cities due to terrain gradients, are key nodes within parks that connect terrain, guide flow lines, promote social interaction and emotional recovery, and accommodate high-frequency social interactions [6,7]. The gathering spaces can effectively improve young adults’ emotional states and generate positive perceptual feedback when properly laid out and designed [8]. Therefore, measuring young adults’ physiological and psychological perceptions of gathering spaces and identifying the primary spatial environmental elements influencing these perceptions can effectively enhance the overall spatial quality of parks and serve as an important avenue for studying the human-environmental conditions and perception mechanisms of mountainous urban parks.

Existing research indicates that, influenced by early recognition, the leisure time of young adults is predominantly occupied by social media and electronic devices, resulting in a persistently low frequency of park visits [9,10]. Given the physiological functions, lifestyles, and individual consciousness inherent to different age groups, significant variations exist in their behavioral preferences and perceptions [11]. Driven by their entertainment and social needs, young adults exhibit a stronger preference for gathering spaces characterized by vibrancy, diverse landscape variations, and pronounced visual and auditory environmental stimuli [12]. Consequently, it is imperative to investigate how spatial characteristics within parks influence the perceptions of this specific demographic. Reviewing current literature, the beneficial effects of urban parks on stress recovery and mood improvement have been clearly documented [13,14,15]. Existing research suggests that soundscape perception in urban parks is closely related to objective environmental elements such as landscape enclosure and aesthetic appeal [16], whereas visual landscape perception is associated with environmental elements such as spatial concealment and naturalness [15,17]. Additionally, as perception is the result of the combined effects of multiple senses [18]. Furthermore, multisensory comfort is an essential contributor to the process of restoration [19]. Specifically, scholars argue that when the characteristics and themes of visual and auditory landscapes are consistent, such as when natural soundscapes like birdsongs and insect chirps are present in natural environments with rich vegetation and high green view index, they are perceived as more effective in regulating user emotions and alleviating stress than single-environment settings [20,21]. In mountainous urban parks, terrain and spatial variations result in variability in both visual and auditory environments [22,23]. Therefore, exploring the impacts of different visual and auditory environments on the perception of various types of gathering spaces is of practical significance.

In psychological research encompassing abstract mental activities such as cognition, emotion, and willpower, existing studies primarily utilize psychological scales such as the Profile of Mood States (POMS) and the Perceived Restorativeness Scale (PRS) [24,25] to measure changes in users’ psychological perceptions in specific urban environments. In terms of physiological research based on sensory organs and neural transmission mechanisms, indicators reflecting physiological perception changes, such as Heart Rate Variability (HRV), Respiratory Rate (RESP), α-EEG (α-electroencephalogram), β-EEG, and Average Pupil Diameter (APD), have been widely used to assess the impact of urban park landscapes on users’ perceptions [21,26,27,28]. In addition, recent studies have employed coupled analyses of psychological and physiological data to conduct more comprehensive analyses of the interactions between the environment and perceptions [29,30].

Research in environmental psychology and cognitive neuroscience indicates that urban environmental elements can exert certain influences on the psychological and physiological states of users [31]. Especially for young adults, as they are in a stage characterized by sensitive emotional reactivity and high emotional plasticity, the perceptual feedback triggered by environmental stimuli presents more complex variation characteristics [32]. For instance, previous studies have highlighted that factors such as vegetation or shading coverage affect comfort perception and significantly influence user behavior in urban spaces [33,34,35]. Ulrich proposed the Stress Recovery Theory (SRT) in 1983, which demonstrates that stress variations are psychological and behavioral responses to the surrounding environment, and that natural environments can elicit positive emotions and promote physiological restoration benefits [36]. Meanwhile, the Attention Restoration Theory (ART) also suggests that visual and auditory elements of nature help regulate autonomic nervous system activity and restore cognitive resources such as attention [37]. The concept of “Shinrin-yoku” proposed by Japanese scholars also demonstrates the potent efficacy of natural environments like forests in mood enhancement, particularly for individuals who have experienced significant stress [38]. However, mountainous areas are characterized by significant topographic relief, complex slope variations, and the resulting three-dimensional urban spatial structure. These features fundamentally lead to differences in their visual and acoustic environments compared to those of plain cities [39]. Specifically, topographic undulations shape dynamically changing visual corridors, while variations in elevation and aspect create viewing points that alter the visual openness of spaces. Simultaneously, the changing elevations in mountainous areas form natural barriers, such as embankments, which reflect and guide sound wave propagation, resulting in highly location-specific perception of the soundscape [22]. These visual and auditory variations are particularly pronounced in complex ecological and topographical settings like mountainous parks [14]. Furthermore, typical spatial indicators including openness, elevation, and concealment shape users’ unique subjective perceptions with mountainous characteristics. For instance, significant elevation variation raises concerns about accessibility, while complex terrain coupled with diverse soundscapes jointly forms the perception of complexity.

To investigate the impact of spatial environments in mountainous urban parks on young adults’ psychological perceptions and physiological responses, at least two primary stimuli that significantly influence young adults must be considered: visual landscapes and soundscapes [40]. However, current research on the different types of gathering spaces in mountainous environments remains relatively limited, with most studies focusing on parks at different altitudes or with distinct functionalities. Although Chen et al. (2023) indicated in their study that different visual landscape elements in mountainous parks may differentially impact young adults’ restorative outcomes, the question of whether auditory and audiovisual interactions could produce distinct effects remains to be further investigated [31]. Gong et al. (2025) preliminarily demonstrated that environmental elements in mountainous conditions can influence visitors’ perceptions [39]; however, their discussion primarily focused on the impact on subjective psychological perceptions. These findings suggest that in the complex spatial environments of mountain parks, the same environmental elements may elicit different perceptual responses from young adults in different spatial contexts. Therefore, classifying gathering spaces based on spatial characteristics could facilitate further research on the mechanisms linking mountain park environments to multi-dimensional perceptions.

Previous studies have often been conducted in laboratory settings to strictly control environmental variables [41,42], analyzing single indicators or the same category of indicators, such as focusing on the impact of space on physiological indicators, or primarily focusing on plain cities. However, due to their unique topographical and geomorphological environments, mountainous areas bring about spatial environmental characteristics and landscape layouts entirely different from those of plain cities. Their perceptual experiences rely on the dynamic coupling of multiple senses, which to some extent influences users’ behavioral activities and spatial decision-making, thereby affecting their perception of the environment [39]. Laboratory environments cannot fully replicate the multisensory conditions of mountain parks, nor can they account for the multi-dimensional perceptual changes influenced by diverse factors such as participants’ behavioural activities and physical exertion [43]. Conducting experiments in the field environment of mountainous parks enables participants to better capture the variations in visual and auditory landscapes within the mountainous setting. From the perspectives of on-site experience and real-time perception, this approach allows for the investigation of the intrinsic factors influencing perception in gathering spaces within mountainous parks. This study integrates spatial exposure data and physiological and psychological data to explore the mechanism of action on users’ perception within this unique spatial environment of mountains.

Against this background, this study, based on stress recovery and attention restoration theories, aimed to identify key subjective perception indicators in typical gathering spaces within mountain urban parks and to investigate which visual and auditory spatial characteristics influence young adults’ psychological perceptions and physiological responses, as well as what perception factors influence young adults’ gathering behaviour in mountain urban parks. Extensive design practice demonstrates that the emergence of mountainous gathering activities and their associated spaces is fundamentally shaped by mountainous terrain and environmental conditions, primarily concentrated in mountain bases, slopes, and summits. Accordingly, based on mountain heights, spatial elements, and functional attributes, this study categorizes gathering spaces into four types: path platform, elevated point, viewing boundary, and key node. Based on the aforementioned research background, we propose the following hypotheses:

**H1.**  *The operational trends of physiological changes among young adults vary across different types of gathering spaces, and significant differences exist in their psychological perceptions. Compared with the key node, young adults’ evaluations of the psychological perception indicator “Aesthetics” in transit-oriented gathering spaces will be relatively lower.*

**H2.**  *Evaluation indicators that significantly reflect mountainous topographic characteristics, such as “Elevation” and “Accessibility,” will constitute the core metrics explaining spatial perception, and the perceptual profiles of different space typologies will exhibit distinctly different distribution patterns within the principal component space constituted by these core metrics.*

**H3.**  *Influenced by typical spatial environmental features, young adults’ psychological perceptions and physiological responses will show significant differences across different spaces; for example, the “Natural Soundscape” will exert a significant influence on physiological indicators such as α-EEG [15]?*

**H4.**  *Natural elements play a significant role in alleviating young adults’ stress perceptions. A congruent, natural audiovisual matching environment will yield significantly more positive psychological and physiological restorative effects on young adults than single natural stimuli alone [44]?*

## 2. Methods

### 2.1. Study Area

**Figure 1:**
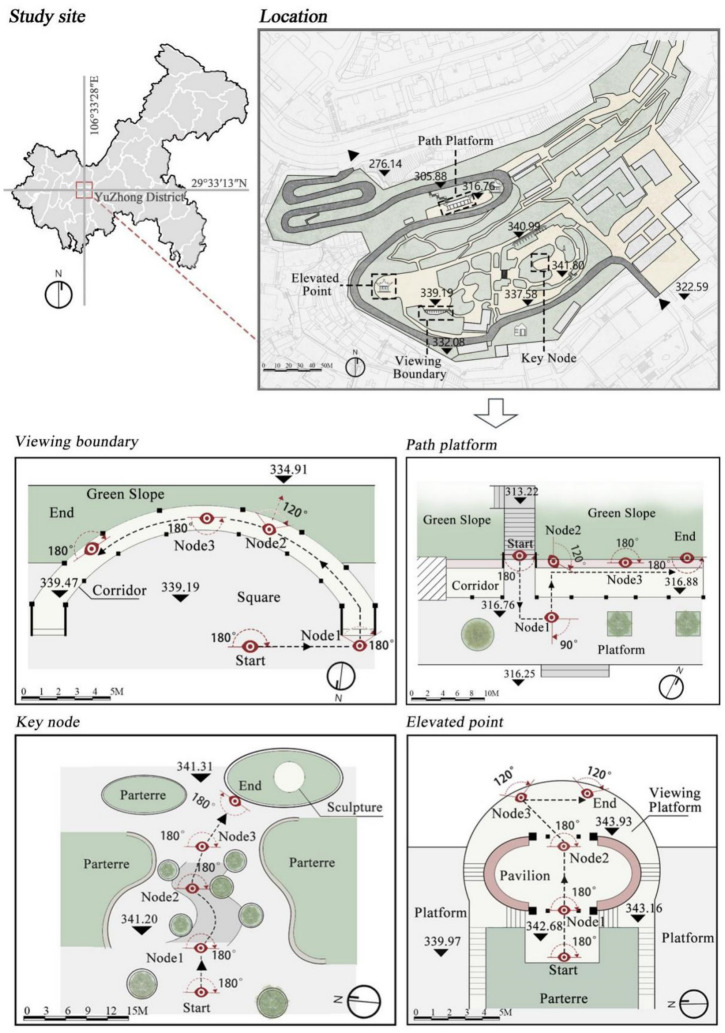
Study Location and Selected Site Profile.

**Figure 2:**
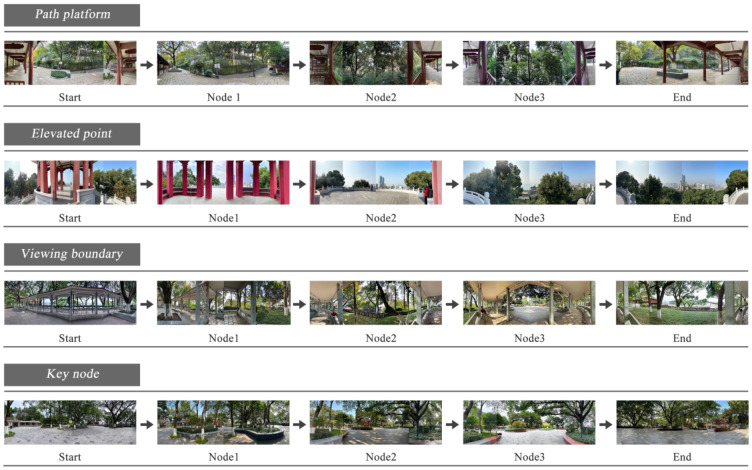
Schematic Diagram of Stop Nodes.

Pipa Mountain Park is located in Yuzhong District, Chongqing City, China, at an altitude of 345 m and an elevation of approximately 70 m, making it one of the highest points in Yuzhong District. It features typical mountainous terrain characteristics, with an annual average temperature ranging from 15 to 23 °C, providing an ideal spatial research subject for human-induced experiments. Through multiple on-site surveys, four types of gathering spaces in Pipa Mountain Park were selected: path platform, elevated point, viewing boundary, and key node (Table 1). Five important stopping nodes were set up in each gathering space to measure the physiological perception feedback of the young adults at that node. Figure 1 and Figure 2 show the locations of the four types of clustered spaces in Pipa Mountain Park, as well as the floor plans, stopping points, and elevation information for the four types of clustered spaces.

### 2.2. Participants

We recruited 48 participants for this experiment (M = 22 years, SD = 1.62, range = 18–24 years), including 26 females and 22 males. Sensitivity analysis was conducted using G*Power 3.1 [45]. The initial analysis indicated that with 48 participants, the test had a power of 0.80, capable of reliably detecting an effect size of at least 0.18 (Cohen’s f) using a repeated-measures F-test within factors. Furthermore, a post hoc power analysis based on the actual experimental data revealed that the study achieved a statistical power (1 − β) of 0.9999862 for key indicators, significantly surpassing the standard threshold of 0.80. This confirms that the sample size of 48 was robust enough to prevent Type II errors in this specific experimental design. None of the participants had a history of cardiovascular, mental, or neurological diseases; were taking long-term prescription medications; nor had an uncorrected or corrected visual acuity of 1.0. All participants had good auditory and visual abilities; had not consumed alcohol prior to the experiment; and were in good physical condition on the day of the experiment. All eligible participants who volunteered to participate in the experiment were fully informed of all experimental procedures and consented to the recording of their electrophysiological, electroencephalogram, and eye-tracking data during the experiment, which was solely for scientific research purposes. The participants could withdraw from the experiment at any time.

### 2.3. Spatial Exposure Data

This study aimed to explore the relationship between psychological perceptions and physiological responses and spatial, visual, and acoustic environmental elements in the four types of gathering spaces in mountainous urban parks. Existing studies have shown that in addition to spatial structural elements such as plants, water features, and elevation differences, the synergistic effects of acoustic and visual environments can also have a certain impact on human psychological perceptions and physiological responses [5,46]. Therefore, the research team selected 6 visual environmental elements, 3 acoustic environmental elements, and 2 spatial structural elements that reflect the characteristics of mountainous environments and may influence human perception and experience. The visual environmental indicators include “openness, green view index, concealment, infrastructure ratio, colour richness, and historical building ratio” [47,48]; the acoustic environmental indicators include the “time ratio of natural, artificial, and mechanical sounds during the stay at each node”; and the spatial structural elements include “elevation and material hardness ratio”. Based on the environmental characteristics represented by each element, they were categorised into four typical environmental features: openness, natural landscapes, natural soundscapes, and elemental complexity (Table 2). The definitions and calculation methods for each element are listed in Table S1. Visual indicator data were obtained through calculations. We set up fixed tripods at a height of 1.5 m at the geometric centre of each node under similar weather conditions, mimicking the horizontal field of view of the human eye to capture panoramic photos. Using Photoshop, they manually delineated the boundaries for each visual factor and calculated the ratio of a factor segment to the entire pixel area, thereby determining the final numerical values for each spatial element in subsequent analyses [31]. A Lotoo PAW-VE high-fidelity recorder was used for audio acquisition, and the audio was normalised using an Adobe Audition digital audio workstation.

### 2.4. Physiological and Psychological Data Processing

To collect the psychological responses of the young adults to the visual and acoustic environments of the four types of gathering spaces, we constructed a subjective evaluation scale suitable for this study by referring to perception indicators widely used in landscape assessment in previous studies [28,49,50,51]. We developed a subjective evaluation scale tailored to this study, wherein all perceptual dimensions were rated on a 7-point Likert scale from −3 (most negative) to +3 (most positive). The scale consists of three sections: personal information and physical activity level, visual environment perception evaluation, and acoustic environment perception evaluation. The perception evaluation section of the scale covers multiple dimensions of the visual environment, including aesthetics, safety, natural elements, comfort, accessibility, social, publicness, infrastructure, complexity, order, culture, recognisability, and elevation, as well as multiple dimensions of the acoustic environment, including pleasantness, anxiety, tranquillity, disorder, vibrant, monotony, eventful, uneventful, natural sounds, and diversity.

To investigate the physiological and perceptual effects of spatial environments on participants, they were required to wear a Tobii Pro Glasses 2 wearable eye tracker (Tobii, Stockholm, Sweden), an ErgoLAB EEG hydroelectrode electroencephalograph (Kingfar International Inc., Beijing, China), an ErgoLAB Ear intelligent wearable ear-clip sensor (Kingfar International Inc.), and an ErgoLAB Bio Sensing human-factor measurement device (including finger sensors and chest strap sensors; Kingfar International Inc.). Physiological perception-related data were collected using the ErgoLAB Human–Machine Environment Synchronisation Platform V3.0 and the ErgoLAB Datalogger APP mobile terminal human factor recording system. Prior to formal testing, the eye tracker underwent a one-point geometric card calibration, while EEG electrode impedance and physiological sensor waveforms were verified via real-time software feedback and the 3 min resting-state trial to eliminate baseline drifts or motion artifacts. Although distinct from clinical medical-grade instruments, the measurement precision and reliability of these mobile wearable systems have been thoroughly validated in field-based environmental psychology and behavioral architecture studies, satisfying the analytical requirements of the empirical model. Building on commonly used indicators selected in existing spatial perception and recovery studies [27,28], and in line with the research objectives and content of this study, physiological electrical indicators such as Respiratory Rate (RESP) and Heart Rate Variability (HRV); EEG indicators including α-EEG (8–13 Hz), β-EEG (14–30 Hz), and β/α; average pupil diameter (APD), fixation frequency (FF), and saccade frequency (SF) were used as indicators reflecting participants’ physiological perception (Table S2). Among these, HRV serves as a quantitative indicator of the dynamic balance between the sympathetic and parasympathetic branches of the autonomic nervous system. LF/HF quantifies the power ratio between the two frequencies, thereby reflecting the participants’ emotional valence and stress perception. Additionally, HR is a direct output of sympathetic activation, enabling real-time quantification of emotional arousal. Therefore, these two indicators were selected as primary research metrics within HRV.

### 2.5. Procedure

**Figure 3:**
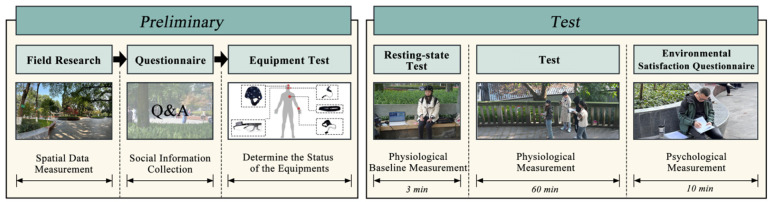
Experimental Procedure.

We conducted a 14-day field experiment in a mountainous urban park between October and November 2023. The experiments were carried out from 10:00 to 16:00 on overcast but rain-free days under relatively stable weather conditions, where temperature ranged from 18 to 23 °C, humidity levels remained between 60% and 80%, and light intensity varied from 1500 to 20,000 lx. Before the experiment, participants were briefed on the entire experimental process and asked about their physical and mental states. After the participants understood and agreed to all terms, they were fitted with the experimental equipment and underwent a 3 min resting-state test. In the four types of gathering spaces, fixed observation routes were established. Stopping points (nodes) were set at the starting and ending points of each space type and at key turning points where visual and acoustic environments changed. Participants walked at a constant speed along the designated route and were required to stop at these nodes to observe for 15 s, following a specified observation angle (180°) and direction. To minimize potential physical fatigue caused by the significant elevation differences in the mountainous terrain, where the vertical height difference between the lowest and highest spaces reaches approximately 25–30 m, a fixed experimental sequence arranged along the shortest climbing path was adopted. While a randomized sequence can theoretically counterbalance order effects, repeatedly ascending and descending steep mountainous terrain would introduce excessive physical exertion and cardiorespiratory confounding effects, severely compromising the baseline validity of the physiological data. The experiment for each type of gathering space took approximately 5–7 min to complete. Prior to proceeding to the next gathering space, participants were required to sit quietly and rest for 8–10 min, during which they completed a ‘subjective evaluation scale’. Each participant completed two repeated experimental sessions. To guarantee data integrity and reproducibility, a rigorous three-tier data quality control screening was applied to select the optimal trial for subsequent analysis. The selection criteria were based on: (1) data collection rate, ensuring optimal real-time wireless sampling efficiency via the ErgoLAB platform; (2) signal waveform stability, prioritizing sessions with distinct periodic physiological waveforms and minimal motion artifacts or baseline drifts; and (3) export integrity, requiring complete and synchronized time-series logs across all multimodal sensors. This screening process minimized potential errors arising from random environmental interference and equipment displacement during field walking. The experimental process is illustrated in Figure 3.

### 2.6. Data Analysis

**Figure 4:**
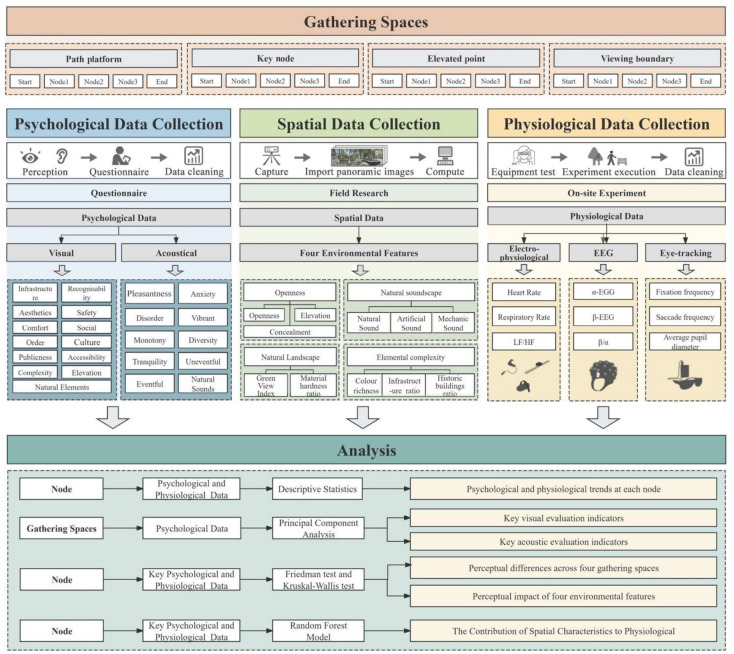
Data Collection and Analysis Framework.

Valid data from 48 participants were pre-processed using ErgoLAB V3.0, yielding 1728 node fragment datasets. All indicators were normalized to reduce baseline physiological variability and facilitate multi-dimensional coupling [52]. Data analysis was performed in R 4.4.1, following the framework in Figure 4. First, descriptive statistics visualized perceptual trends and physiological signal dynamics across the four space types. Second, Principal Component Analysis (PCA) with Varimax rotation reduced 23 perception indicators into key components, using a 0.30 factor loading threshold as the standard minimum interpretable cut-off for exploratory perception data. Third, Friedman and Kruskal–Wallis tests examined significant differences in young adults’ responses across space typologies and environmental levels, with Kendall’s W calculated as the effect size to complement statistical significance, and Pearson correlation analysis was used to further examine whether the physiological indicators changed independently or showed coupled stress-arousal-attention responses. Finally, a random forest model integrated with SHapley Additive exPlanations (SHAP) values was constructed to quantify the contribution and directional effects of specific spatial elements and psychological indicators on physiological perception, with Grid SearchCV and 5-fold cross-validation implemented to explicitly mitigate overfitting risks and ensure model robustness.

## 3. Results

### 3.1. Descriptive Statistics

**Figure 5:**
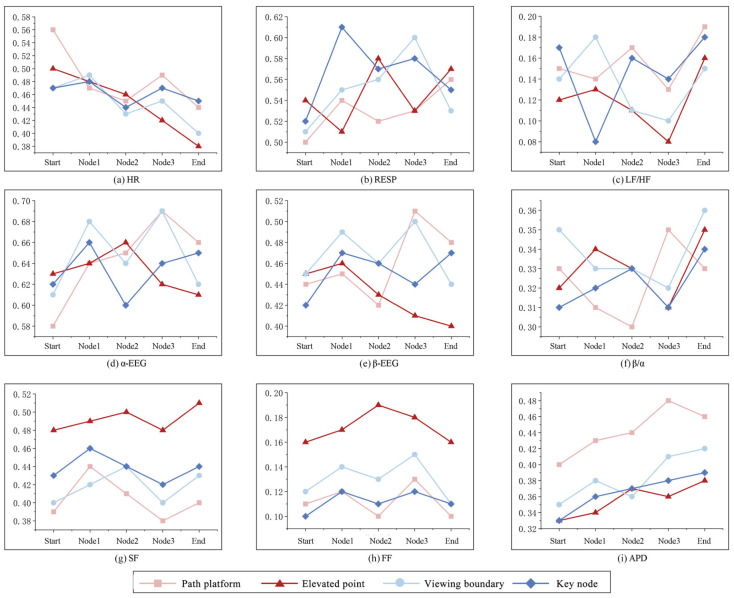
Descriptive statistics of Physiological Indicators data. Note: All physiological indicators are normalized dimensionless values; the x-axis represents the sequential observation points along the experimental route.

The physiological indicators measured at the start, Node 1, Node 2, Node 3, and end of the experimental path were used as time variables to statistically describe the changing trends of psychological, electroencephalographic, and eye movement indicators in the four types of gathering spaces, and were plotted as a line graph (Figure 5). The results show that in the path platform, HR showed an overall downward trend, β/α showed a significant increase at Node 2 and then gradually decreased. In the elevated point, LF/HF reached its lowest point at Node 3 and then rose slightly, while β-EEG showed a gradual downward trend, and the values of SF and FF were higher than the other three types of gathering spaces throughout the experiment. In the viewing boundary, the trend of HR was similar to that of other gathering spaces with β/α values relatively low in Node 1 to Node 2, although the changes in EEG data were relatively stable, and APD showed a gradual upward trend. In the key node, LF/HF showed a very significant decline in Node 1 and gradually rose after reaching its lowest point, whereas α-EEG rose slowly from start to finish, and eye movement indicators showed a similar trend to the other three types of gathering spaces.

A total of 48 questionnaires were collected for the experiment. The reliability and validity of the 48 questionnaires were tested (KMO > 0.6, Bartlett’s sphericity test *p* < 0.001), with Cronbach’s α coefficient of 0.81 confirming internal consistency reliability, and the results indicated that the data were valid. Statistical analyses were performed using data from 23 psychological perception indicators. The results (Table 3 and Table 4) showed that in the path platform, the visual evaluation indicators ‘complexity’ (M = 1.51, SD = 0.94) and ‘comfort’ (M = 1.39, SD = 0.91) received high evaluations, and the SD values were both less than 1, indicating that the participants’ evaluations were relatively consistent. In the auditory evaluation indicators, ‘pleasantness’ (M = 0.84, SD = 1.52) and ‘monotony’ (M = 0.92, SD = 1.13) received higher evaluations, although there were significant differences in evaluations among participants. In the elevated point category, the visual evaluation indicator ‘natural elements’ (M = 0.10, SD = 1.48) received lower evaluations. Among the auditory evaluation indicators, except for ‘diversity’ (M = 0.84, SD = 1.52), evaluations of other indicators were relatively concentrated, with the indicator ‘pleasantness’ having the highest evaluations (M = 1.55, SD = 1.04). In the viewing boundary, participants’ evaluations of auditory indicators were generally positive, with the highest evaluations for ‘pleasantness’ (M = 0.82, SD = 1.01) and ‘vibrant’ (M = 0.88, SD = 1.09). In the key node category, the evaluations of ‘publicness’ (M = 1.71, SD = 0.94), ‘safety’ (M = 1.49, SD = 0.82), and ‘order’ (M = 1.35, SD = 0.69) were relatively high, with smaller standard deviations.

### 3.2. Psychological Perception Evaluation Based on PCA

PCA was employed to extract the first five principal components with eigenvalues greater than one from the visual and auditory environment evaluation metrics of psychological perception. A Varimax rotation was applied to the component loading matrix (Table 5 and Table 6) to identify and screen the key evaluation indicators affecting the participants’ perceptions of the four types of gathering spaces in mountainous urban parks. The results indicate that in the visual environment evaluation indicators, the variance explained by Principal Component 1 was 25.22%, with the indicators ‘comfort’ and ‘social’ loadings the most (0.719, 0.652). The variance explained by Principal Component 2 was 12.97%, with the highest loadings from ‘elevation’ and ‘accessibility’ (0.651, 0.650). Since these four indicators had a significant impact on the visual environment evaluation, they were selected as key visual environment indicators. In the acoustic environment evaluation indicators, the variance explained by Principal Component 1 was 31.45%, with ‘uneventful’ and ‘vibrant’ having the highest loadings (0.794, 0.671). The variance explained by Principal Component 2 is 23.12%, with ‘disorder’ and ‘tranquillity’ having the highest loadings (0.818, −0.727). Therefore, these four indicators were selected as key acoustic environment indicators in the acoustic environment evaluation.

**Figure 6:**
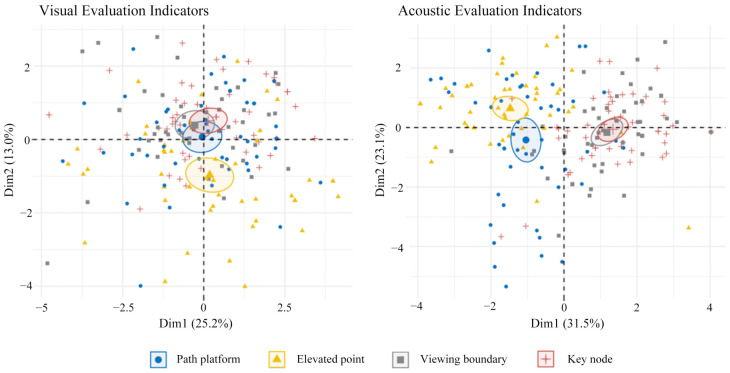
Biplot of principal components for visual–acoustic evaluation indicators.

The PCA biplot results, categorized by the four types of gathering spaces (Figure 6), reveal distinct patterns in the psychological perception of visual environment indicators. The Path Platform exhibits a relatively concentrated distribution in the principal component space. In contrast, the Elevated Point is situated in the negative quadrant of both Principal Component 1 and Principal Component 2, indicating a relatively lower comprehensive perception score in this space, as constituted by indicators such as “comfort,” “social,” “elevation,” and “accessibility.” However, the viewing boundary and key node are generally oriented toward the positive quadrant. In the psychological perception of the acoustic environment evaluation indicators, path platform scores are primarily distributed in the negative quadrant of Principal Component 1, indicating that its evaluation of the space leans toward ‘monotony’; Elevated Point is distributed across both the negative quadrant of Principal Component 1 and the positive quadrant of Principal Component 2, indicating that while the space lacks a certain degree of vitality, its complexity effectively improves participants’ psychological perceptions. The viewing boundary and key node are primarily distributed in the positive quadrant of Principal Component 1, indicating a high degree of consistency in psychological perceptions.

### 3.3. Psychological and Physiological Differences in Perception

#### 3.3.1. Differences in Psychological and Physiological Indicators

The study further selected HR, α-EEG, β-EEG, and APD as physiological indicators that can intuitively and comprehensively reflect stress recovery and environmental arousal, combined with the key visual and auditory psychological perception indicators derived from principal component analysis. The Friedman test was used to investigate whether there were differences in the psychological and physiological perception data in the four types of gathering spaces. The results (Table 7) showed that among the physiological indicators, APD (χ^2^ = 9.375, *p* = 0.000), α-EEG (χ^2^ = 17.096, *p* = 0.001) and β-EEG (χ^2^ = 9.759, *p* = 0.001) showed significant differences (*p* < 0.05) among the four types of gathering spaces, with APD having the most significant. However, there were no significant differences in the HR among the different spaces (*p* > 0.05). To further determine whether these physiological responses represented independent changes or a coordinated process, Pearson correlation analysis was conducted among the four physiological indicators (Figure S1). The results showed a moderate positive association between α-EEG and β-EEG (r = 0.349, *p* < 0.001), and weak but significant positive associations between β-EEG and APD (r = 0.132, *p* < 0.001) and between α-EEG and APD (r = 0.089, *p* = 0.006). HR was not significantly correlated with α-EEG, β-EEG, or APD (all *p* > 0.05), indicating that the EEG and eye-movement indicators were more closely coupled than HR in the field experiment. Among the psychological indicators, except for ‘social’, which showed no significant differences among the four types of gathering spaces (*p* > 0.05), there were significant differences among the four types of gathering spaces for all other indicators (*p* < 0.05). Among these, the differences in ‘vibrant’ (χ^2^ = 65.963, *p* < 0.001) and ‘uneventful’ (χ^2^ = 37.457, *p* < 0.001) were the most significant.

#### 3.3.2. Effects of the Four Spatial Typological Features on Physiological and Psychological Indicators

The results of the Kruskal–Wallis test, which analysed the differences in physiological and psychological indicators of the four types of gathering spaces, preliminarily showed that different types and functions of spaces in mountainous urban parks have a significant impact on the perceptions of the participants. Therefore, based on the four typical environmental characteristics of ‘openness’, ‘natural soundscape’, ‘natural landscape’, and ‘elemental richness’, the four types of gathering spaces were assigned values and graded (Table 8), and the Kruskal–Wallis test was used to investigate whether physiological and psychological perceptions differed significantly according to the levels of the four typical environmental characteristics. The results indicate (Table S3) that under the influence of ‘openness’, HR (χ^2^ = 15.001, *p* < 0.05) showed significant differences at the 0.05 level, while all other physiological and psychological perception indicators exhibited significant differences (*p* < 0.01). Under the influence of ‘natural soundscape’, physiological indicators α-EEG (χ^2^ = 23.728, *p* < 0.01) and APD (χ^2^ = 99.232, *p* < 0.01), as well as all psychological indicators, showed significant differences, while HR did not show significant differences under the influence of this spatial characteristic. Under the influence of the ‘natural landscape’, HR showed no significant differences, and the significance of the psychological indicator social (χ^2^ = 8.977, *p* < 0.05) was relatively weak. Under the influence of ‘elemental complexity’, APD (χ^2^ = 68.428, *p* < 0.01) and the psychological indicators ‘vibrant’ (χ^2^ = 163.674, *p* < 0.01) and ‘uneventful’ (χ^2^ = 127.990, *p* < 0.01) showed significant differences in the four types of gathering spaces.

### 3.4. Impact of Spatial Elements on Physiological Perception

#### 3.4.1. Random Forest Model

To further quantify these impacts, a random forest model was established to explore the impact of specific spatial indicators on perception in the four types of gathering spaces. The eleven spatial element indicators contained in the four types of gathering spaces and the key psychological indicators based on principal component analysis were used as independent variables, whereas the physiological indicator data were used as the dependent variable. A total of 192 samples from 48 participants in the four types of gathering spaces were divided into training and test sets at a ratio of 4:1, and a Grid SearchCV was used to optimise the model parameters and select the best parameter combination. K-Fold Cross-Validation was used to assess the model reliability. The model was trained using the optimal parameters, with the coefficient of determination (R^2^), root mean square error (RMSE), mean absolute error (MAE), and mean bias error (MBE) serving as the evaluation metrics (Equations (1)–(4)). The results showed that there were differences in the performances of the models for the four types of gathering spaces in mountainous urban parks in the training and test sets (Table S4). The APD showed a high degree of interpretability in all four types of gathering spaces and was highly sensitive to changes in spatial characteristics. α-EEG and β-EEG performed better in the elevated point and key node than in the path platform and viewing boundary, possibly due to factors such as accessibility in the latter two causing interference with participants’ perceptions in mountainous urban parks. This conclusion was further validated by a feature importance analysis.

(1) $$R^{2}=1-\frac{\sum_{i=1}^{n}{\left(y_{i}-{\hat{y}}_{i}\right)}^{2}}{\sum_{i=1}^{n}{\left(y_{i}-{\bar{y}}_{i}\right)}^{2}}$$

(2) $$RMSE=\sqrt{\frac{\sum_{i=1}^{n}{\left(y_{i}-{\hat{y}}_{i}\right)}^{2}}{n}}$$

(3) $$MAE=\frac{\sum_{i=1}^{n}\left|y_{i}-{\hat{y}}_{i}\right|}{n}$$

(4) $$MBE=\frac{\sum_{i=1}^{n}\left(y_{i}-{\hat{y}}_{i}\right)}{n}$$

where $y_{i}$ represents the actual observed physiological baseline value of participant *i*, ${\hat{y}}_{i}$ represents the predicted value by the random forest model, ${\bar{y}}_{i}$ is the mean of the actual observed values, and *n* represents the total number of samples (*n* = 192).

#### 3.4.2. Contribution of Spatial Characteristics to Physiological Indicators

**Figure 7:**
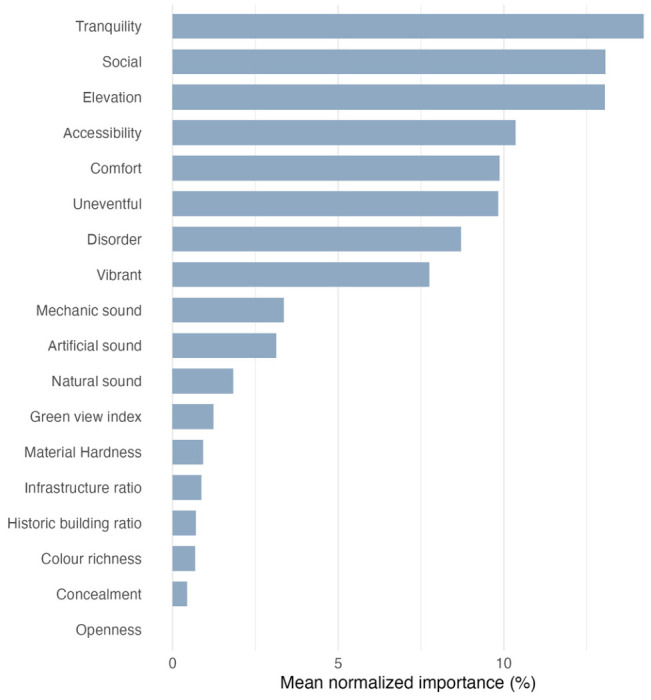
Random forest feature importance ranking.

Random forest feature importance ranking was performed on the four types of gathering spaces as a whole, and the results indicate (Figure 7) that tranquility (14.23%), social (13.07%), and elevation (13.04%) are the primary predictors. The marginal-effect analysis showed that increasing elevation from the 25th to the 75th percentile changed predicted HR by −0.077, β-EEG by +0.026, and APD by −0.049. Over the same percentile range, green view index reduced predicted HR, α-EEG, β-EEG, and APD by −0.004, −0.004, −0.005, and −0.002, respectively; natural sound reduced them by −0.008, −0.003, −0.001, and −0.004, respectively.

**Figure 8:**
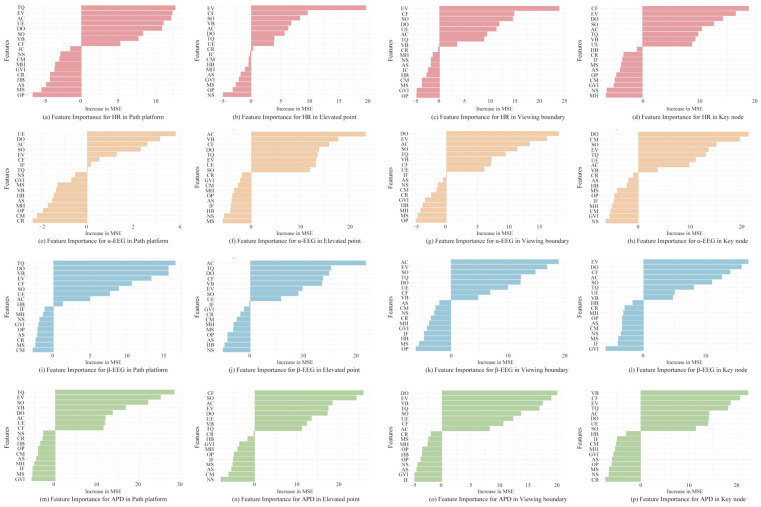
Impact of spatial elements on physiological indicators based on Shapley Additive Explanations (SHAP) analysis. Note: TQ: Tranquility; EV: Elevation; AC: Accessibility; UE: Uneventful; DO: Disorder; SO: Social; VB: Vibrant; CF: Comfort; IC: Infrastructure ratio; NS: Natural sound; CM: Concealment; MH: Material Hardness; GVI: Green view index; CR: Colour richness; HB: Historic building ratio; AS: Artificial sound; MS: Mechanic sound; OP: Openness.

The feature importance metric (%IncMSE) in the random forest model was calculated using SHAP analysis. The specific effects of spatial and key psychological indicators on the participants’ physiological responses were quantified by calculating the contribution values of the features and their directional impacts on model predictions. The results (Figure 8) show that, among the visual environment indicators, elevation has a greater impact on physiological responses in the four types of gathering space models. In the elevated point, the feature importance of elevation on HR was 19.51%; in the path platform, the feature importance of elevation on APD reached 24.99%. Moreover, the greater the elevation, the higher the subject’s HR and APD. In the four types of gathering spaces, increases in openness and green view index significantly reduced HR, β-EEG, and APD, and the explanatory power of openness and green view index on HR was higher in the path platform and key node than in the other two types of spaces. Regarding acoustic environment indicators, natural sounds and artificial sounds have a more significant reducing effect on β-EEG in the elevated point and key node compared to the path platform and viewing boundary. At the same time, the SHAP analysis results show that among the four types of gathering spaces, the key psychological indicator ‘social interaction’ has a positive effect on α-EEG, that is, the higher the ‘social’ indicator score, the higher the α-EEG. At the same time, ‘disorder’ has a promoting effect on β-EEG, and ‘vibrant’ has a promoting effect on APD. When the ‘infrastructure’ score increases, the participants’ HR decreases accordingly. The results of further interaction analysis indicate that the interaction between the green view index and natural sound factors has a significant effect on the β-EEG indicator (βstd = 0.163, *p* < 0.001).

## 4. Discussion

### 4.1. Changes and Differences in Psychological and Physiological Indicators

Descriptive statistics of the collected psychological and physiological data showed that in terms of overall trends, HR and APD showed similar results, with HR showing a gradual downward trend and APD showing a gradual upward trend. The psychological perception visual evaluation indicators ‘aesthetic’ and ‘publicness’ and the auditory evaluation indicators ‘pleasantness’ and ‘comfort’ were relatively high for all four types of gathering spaces. A sub-space analysis revealed that compared to the path platform, viewing boundary, and key node, elevated points—spaces with higher openness, natural elements, and a larger proportion of natural sounds—were associated with stronger restorative feedback on stress relief and emotional recovery in young adults [46,53], as reflected by reductions in HR and β-EEG. Meanwhile, the values of eye movement metrics such as fixation frequency and saccade frequency were significantly higher in the elevated point than in the other three gathering spaces, with young adults’ psychological perception ratings for “aesthetic” and “comfort” also being higher. This indicates that the open vistas at the elevated point provide richer visual stimulation. As corroborated by Taczanowska et al. (2024) [12], young adults exhibit higher demands for visual environmental diversity and tend to favor personalized environmental interaction experiences. Additionally, descriptive statistics revealed that the RESP of the path platform showed an overall upward trend, whereas the key node exhibited the opposite trend, with a significant decline after Node 1. Furthermore, the APD of the key node was significantly lower than that of the path platform throughout the experiment. This may be because the key node primarily consists of natural landscapes, whereas the path platform predominantly features hard spaces, such as corridors and roads. Nilssion et al. (2010) demonstrated that hard or built spaces do not significantly alleviate young adults’ perceived stress compared to natural landscapes [54]. Research by Nilssion et al. (2010) has demonstrated that, compared to natural landscapes, hard or built spaces tend to be more monotonous and characterized by higher enclosure [54]. Such environments can trigger negative emotions in young adults and fail to significantly alleviate their perceived stress [55]. This also explains why the key node’s ‘aesthetic’ and ‘pleasantness’ ratings were significantly higher than those of the path platform in the audiovisual perception evaluation.

### 4.2. Key Indicator Differences in Mountain Characteristics Among Four Types of Gathering Spaces

Our findings revealed that indicators related to mountainous spatial characteristics are key indicators of psychological perception. In the path platform, the young adults’ evaluations of the visual environment showed a concentrated distribution of scores, and their evaluations of the acoustic environment tended to be ‘monotonous,’ indicating that gathering spaces dominated by functionality lack a certain degree of spatial hierarchy and acoustic variation, which has a negative impact on the young adults’ psychological perception. Meidenbauer et al. (2020) found that spaces lacking hierarchical visual changes and acoustic dynamicity may lead to reduced recovery benefits of young adults, further supporting this conclusion [15]. Additionally, research has shown that when environmental stimuli fall below the cognitive engagement threshold, overactivity in the default mode network can cause young adults to perceive the space as monotonous and uninteresting [56]. By contrast, in spaces with relatively complex audiovisual environments, such as elevated point, evaluations of ‘comfort’ and ‘sociality’ did not show a significant positive advantage. However, the young adults also perceived the sound environment as having a certain level of vitality, indicating that the visual advantages brought about by the elevation changes did not fully translate into positive perceptions. This may be due to the poor accessibility of the elevated point, which could trigger potential stress responses despite their landscape resource advantages [57]. However, an energetic acoustic environment synergistically combined with the visual environment can provide young adults with certain stress recovery benefits [58]. Therefore, there may be differences in psychological perceptions among the young adults in the elevated point.

### 4.3. Differences in Psychological Perceptions and Physiological Responses of the Four Typical Environmental Characteristics

In mountainous urban parks, different environmental characteristics and gathering spaces were associated with significant heterogeneity in user perceptions. Openness and natural landscapes were associated with significant differences in the β- and α-EEGs of young adults in the different gathering spaces. This result, to a certain extent, reveals a visual-dominant mechanism in spatial perception. It aligns with existing research indicating that, compared to other demographics, young adults’ perception of the environment and their subsequent behavioral decisions under environmental stimuli are more heavily dependent on visual information [59]. Natural soundscapes had a more significant effect on α-EEG and APD. In mountainous environments, terrain has a significant impact on soundscape changes; therefore, the perceptions of young adults in different gathering spaces also show certain differences [14]. Elemental complexity was significantly associated on all psychological and physiological indicators, especially α-EEG, APD, and the psychological indicator ‘vibrant’. This may be explained from the perspective of the attention restorative theory [37,60]: when environmental elements within the field of vision are fascinating, such as a sequence of explorable micro-spaces created through the strategic combination of topography and vegetation, they can prompt interaction between young adults and the environment, effectively capturing their interests, and thereby reducing the cognitive load. The relatively weak differences in HR across the four types of gathering spaces may be because HR not only reflects the perception of the spatial environment but is also influenced by emotional responses and stress levels. Complex terrain and microclimate changes in mountainous environments can easily cause anxiety or tension in young adults who are fatigued [61], leading to changes in HR due to the interaction of multiple factors, resulting in a spatial environment with no significant effect.

### 4.4. Impact of Visual and Acoustic Environmental Indicators on Physiological Perception

The SHAP results further revealed a relationship between the spatial characteristics of mountain parks and physiological perceptions. However, given the modest R2 values observed in certain models, the SHAP analysis primarily reflects the relative importance and directional influence of spatial features. The impact of the same spatial elements on the young adults’ perceptions may depend on their proportion in the gathering space. A higher openness of elevated point and key node may help reduce the cognitive load of young adults and lower their APD. Similarly, green view index and natural sound, as primary natural environmental elements in parks, align with previous research confirming the positive significance of green environments in individual restorative experiences [62,63,64,65]. Furthermore, as emphasized by Kemperman and Timmermans (2006) [66], young adults exhibit a distinct preference for green parks, especially those with vibrant colors, due to the increased vitality and visual stimulation they provide. In this study, while higher elevation in the Viewing Boundary and Key Node significantly increased young adults’ β-EEG, a higher proportion of natural elements was found to simultaneously offset this effect. However, it is worth noting although increased openness and natural elements may provide positive perceptual feedback to young adults, their interactions with different environmental elements may yield varying benefits. Therefore, in spaces dominated by natural elements, priority should be given to preserving and introducing natural soundscapes that harmonize with the visual environment to enhance restorative benefits [22]. In contrast, in relatively enclosed spaces primarily serving transportation functions, moderately introducing vibrant artificial soundscapes, such as soft background music or socially generated sounds, can effectively enhance the vitality of the environment and stimulate positive emotional responses in young adults [67]. This strategy finds support in our findings: in the key node, where functional diversity and audiovisual elements are rich, the natural soundscape synergizes with colorful natural scenery and visual landmarks such as landscape sculptures. For example, bird songs appear in environments with high green visibility, and the visual and auditory environments are consistent, which significantly enhances the restorative benefits compared with the other three types of gathering spaces [15,21]. The above conclusions indicate that the perception of the environment is the result of the integration of different types of sensory information. Spatial structural elements can bring positive impacts to young adults’ perception but may also increase perceived stress. Therefore, in the design of gathering spaces in mountainous urban parks, it is essential to reasonably utilize the spatial topography and comprehensively consider the synergistic effects of visual and acoustic landscapes. This involves using combinations of foreground and background to form richly varied landscape layers, integrated with soundscapes to create engaging audiovisual focal points. The audiovisual interaction result suggests that natural visual–acoustic matching should be understood as a conditional physiological modulation mechanism rather than a universally stronger subjective restorative effect.

### 4.5. Design Implications

Based on the aforementioned research results, the design of gathering spaces in mountain parks needs to reasonably utilize the mountainous terrain and balance audiovisual environmental elements. For the path platform, since it primarily bears transit functions, vegetation buffer zones, small resting areas, and soft material transition surfaces can be set up on the basis of predominantly hard paving to reduce participants’ tension, while the artificial sound brought by the resting areas can improve the fit between function and environment to a certain extent, effectively stimulating positive emotions. For the elevated point, open views and natural visual–audio soundscape resources should be appropriately preserved, while utilizing elevation differences to reasonably arrange landscape combinations and introducing natural soundscapes such as the sound of falling water, to avoid the topographical advantages from being affected by negative environmental elements. For the viewing boundary, a layered foreground–background plant configuration and a controlled line-of-sight opening design should be adopted to both maintain visual attraction and avoid excessive enclosure or visual fatigue. For the key node, natural sounds, landmark elements, and moderate social activities can be integrated to enhance spatial vitality, while the interference of mechanical noise needs to be controlled at the same time. Therefore, in the design of gathering spaces in mountainous urban parks, it is essential to reasonably utilize the spatial topography and comprehensively consider the synergistic effects of visual and acoustic landscapes. This involves using combinations of foreground and background to form richly varied landscape layers, integrated with soundscapes to create engaging audiovisual focal points.

### 4.6. Limitations

This study has several limitations. First, the participants were limited to the 18–24 age group. While this demographic represents populations experiencing significant stress [68], perceptual preferences may vary by background and gender, and age differences could further influence responses to environmental stimuli. Second, as the experiment was conducted on-site in a complex mountain environment, it was susceptible to confounding factors beyond the controlled variables. Furthermore, due to topographical constraints, a fixed experimental sequence was employed, which may have introduced ordering effects. Additionally, while selecting the trial with superior signal quality ensured data usability, omitting the alternative dataset may underestimate intra-individual variance and introduce subtle selection bias, potentially limiting overall data integrity and strict reproducibility. To address these limitations, future research should consider the impact of environmental hysteresis on perception, increase sample sizes, and incorporate diverse factors such as socioeconomic status to better capture perceptual diversity. Moreover, future studies could integrate laboratory settings to strictly control extraneous variables and consider comparing forward with reversed sequences or employ randomized sequences to obtain more precise results. Finally, expanding the research scope to encompass more diverse environmental conditions and seasonal variations would provide a more comprehensive foundation for optimizing gathering spaces in mountainous urban parks.

## 5. Conclusions

This study considered four types of gathering spaces in Chongqing’s Biwa Mountain Park as its research objects. Through onsite research, four typical environmental characteristics were identified. The following conclusions were drawn:

(1) There were significant differences in the trends of young adults’ psychological perceptions and physiological responses in different gathering spaces. The elevated point provides superior stress recovery benefits owing to its high openness and natural soundscape. In comparison, the psychological comfort experienced by participants in the path platform, which is primarily characterized by hard landscape features, is relatively lower.
(2) Indicators reflecting the characteristics of mountainous spaces are key indicators of psychological perception, and terrain-driven spatial characteristics can significantly affect the psychological perception of the young adults.
(3) As hypothesized in H3, there were significant differences in psychological perceptions and physiological responses among the four types of gathering spaces. Elemental complexity can effectively attract the attention of young adults, enhance their perception of ‘vibrant’, and elicit positive perceptual feedback.
(4) The synergistic effect of audiovisual environments can enhance the restorative benefits of these spatial elements to some extent; interaction analysis quantitatively demonstrates that the interaction between the green view index and natural sound factors exerts a significant effect on the β-EEG indicator. Furthermore, as hypothesized in H4, natural elements alleviated participants’ stress perceptions. However, similarly, as exemplified by the poor accessibility in the Elevated Point, it can also offset landscape advantages to some extent; therefore, appropriate multisensory integration strategies should be implemented in conjunction with mountainous terrain.

In the future, the optimization and design of gathering spaces in mountainous urban parks should translate these perceptual findings into differentiated spatial strategies: preserving open and natural audiovisual resources in the elevated point, softening hard transit-oriented path platforms, strengthening layered visual organization in the viewing boundary, and coordinating landmark, vegetation, and soundscape elements in the key node. Such strategies can help young adults achieve better psychological recovery and emotional regulation while maintaining accessibility and safe movement in mountainous terrain.

## Figures and Tables

**Table 1:** Spatial characteristics of gathering spaces.

Type	Visual Environmental Features	Acoustic Environmental Features	Elevation	Illustration
Path platform	Dual function of rapid passage and resting; rich in colour; dominant feature is hard paving	The acoustic environment is dominated by anthropogenic sounds such as human conversation.	316.86 m	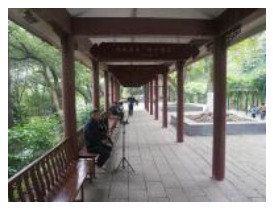
Elevated point	Dual value of elevated view and historical culture; highest degree of openness	The acoustic environment is dominated by natural sounds such as birdsong.	343.93 m	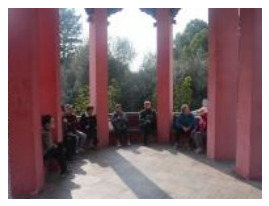
Viewing boundary	Highly representative of mountainous settings; cohesive social space; relatively high openness; diverse in colour and material	The acoustic environment is composed primarily of both anthropogenic and natural sounds.	339.19 m	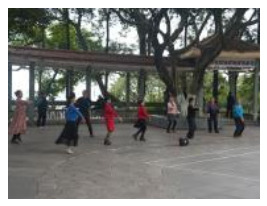
Key node	Representative space combining social and natural functions; diverse activity types; high openness and green view index	The acoustic environment is diverse, comprising anthropogenic, natural, and mechanical sounds.	340.98 m	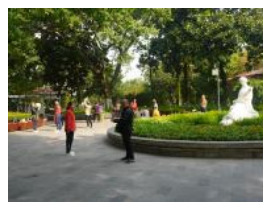

**Table 2:** Classification of Spatial Indicators into Four Typical Environmental Features.

Typical Environmental Feature	Definition	Included Spatial Indicators
Openness	Reflects the degree of spatial exposure and vertical topography	Openness
		Elevation
		Concealment
Natural soundscape	Characterizes the composition of auditory environments with natural dominance	Natural sound
		Artificial sound
		Mechanic sound
Natural landscape	Characterizes natural elements and material softness in visual environments	Green view index
		Material hardness ratio
Element complexity	Measures visual diversity and built-environment layers	Colour richness
		Infrastructure ratio
		Historic buildings ratio

**Table 3:** Statistics of visual evaluation indicators.

		Aesthetics	Safety	Comfort	Natural Elements	Elevation	Accessibility	Social	Infrastructure	Publicness	Complexity	Order	Culture	Recognisability
Path platform	M	1.27	0.94	1.39	0.57	0.16	0.94	1.22	0.78	1.37	1.51	1.27	1.20	0.82
	SD	0.95	1.30	0.91	1.40	1.83	1.27	1.09	1.25	1.13	0.94	0.91	0.79	1.48
Elevated point	M	1.90	1.37	1.63	0.37	0.71	0.59	1.22	0.02	0.90	1.41	1.33	1.37	0.96
	SD	0.98	1.17	1.09	1.52	1.67	1.14	1.34	1.03	1.45	1.21	1.28	1.20	1.63
Viewing boundary	M	1.29	1.10	1.22	0.31	−0.18	1.04	1.04	0.86	1.47	1.31	1.04	0.49	1.29
	SD	0.94	0.98	0.90	1.49	1.67	1.12	1.10	1.08	1.26	1.16	1.10	1.61	1.19
Key node	M	1.47	1.49	1.20	0.92	−0.47	1.27	1.22	0.88	1.71	1.02	1.35	0.51	1.14
	SD	0.79	0.82	0.96	1.38	1.85	1.08	1.12	1.33	0.94	0.99	0.69	1.50	1.17

**Table 4:** Statistics of acoustical evaluation indicators.

		Pleasantness	Anxiety	Tranquility	Disorder	Vibrant	Monotony	Eventful	Uneventful	Natural Sounds	Diversity
Path platform	M	0.84	0.59	−0.29	−0.47	−0.20	0.92	−0.69	0.45	−0.61	−0.27
	SD	1.52	1.43	1.40	1.71	1.14	1.13	1.10	1.12	1.15	1.66
Elevated point	M	1.55	1.31	−0.90	0.43	−0.82	0.84	−0.71	0.35	−0.31	0.10
	SD	1.04	1.04	1.14	1.17	1.03	1.03	1.08	1.15	1.25	1.48
Viewing boundary	M	0.82	−0.80	0.16	−0.47	0.88	−0.76	0.49	−0.27	0.24	0.78
	SD	1.01	1.22	1.50	1.31	1.09	0.99	1.19	1.34	1.36	1.46
Key node	M	0.90	−0.80	0.00	−0.55	1.12	−0.86	0.49	−0.51	0.18	0.88
	SD	0.96	1.08	1.54	1.29	0.86	1.08	1.19	1.29	1.70	1.13

**Table 5:** Rotated component matrix for visual evaluation indicators.

Evaluation Item	Component				
	1 (25.22%)	2 (12.96%)	3 (9.66%)	4 (8.45%)	5 (7.45%)
Comfort	0.719	-	-	-	-
Social	0.652	-	-	-	-
Aesthetics	0.585	-	-	-	-
Order	0.533	-	-	-	-
Culture	0.529	-	-	-	-
Elevation	-	−0.651	-	-	-
Accessibility	-	0.650	-	-	-
Complexity	-	0.481	-	-	-
Safety	-	-	0.448	-	-
Infrastructure	-	-	0.453	-	-
Publicness	-	-	-	0.453	-
Recognisability	-	-	-	0.325	-
Natural Elements	-	-	-	-	0.620

Note: Factor loadings < 0.30 were suppressed for clarity.

**Table 6:** Rotated component matrix for acoustical evaluation indicators.

Evaluation Item	Component				
	1 (31.45%)	2 (23.12%)	3 (13.24%)	4 (7.61%)	5 (6.51%)
Uneventful	0.794	-	-	-	-
Vibrant	0.671	-	-	-	-
Monotony	−0.622	-	-	-	-
Disorder	-	0.818	-	-	-
Anxiety	-	0.693	-	-	-
Tranquility	-	−0.727	-	-	-
Pleasantness	-	-	0.977	-	-
Diversity	-	-	0.329	-	-
Natural Sounds	-	-	-	0.805	-
Eventful	-	-	-	-	0.693

Note: Factor loadings < 0.30 were suppressed for clarity.

**Table 7:** Friedman Test Results.

Indicator		Chi-Square	*p*-Value	Effect Size (Kendall’s W)
Psychological	Comfort	8.873	0.031	0.062
	Accessibility	12.851	0.005	0.089
	Tranquility	13.271	0.004	0.092
	Uneventful	37.457	0.000	0.260
	Disorder	17.398	0.005	0.121
	Social	0.576	0.902	0.004
	Vibrant	65.963	0.000	0.458
	Elevation	14.642	0.002	0.102
Physiological	HR	0.976	0.807	0.007
	α-EEG	17.096	0.001	0.119
	β-EEG	9.759	0.021	0.068
	APD	9.375	0.000	0.065

**Table 8:** Value assignment of gathering spaces.

	Openness	Natural Soundscape	Natural Visual Landscape	Elemental Complexity
Path platform	2	3	2	3
Elevated point	3	2	1	1
Viewing boundary	1	2	3	1
Key node	2	1	2	2

## Data Availability

The data presented in this study are available on request from the corresponding author. The data are not publicly available due to the information concerning the participants.

## Supplementary Materials

The following supporting information can be downloaded at: https://www.mdpi.com/article/10.3390/s26134177/s1, Figure S1: Pearson Correlation Analysis of Physiological Indicators; Table S1: Spatial visual and acoustic indicators; Table S2: Description of Physiological, EEG, and Eye-Tracking Indicators; Table S3: Kruskal–Wallis Test Results; Table S4: Training set and test set results of random forest.
