# Supplementary material for: How Mountain Park Spatial Environments Affect Physiological and Psychological Perceptions of Young Adults Based on Real Time Sensor Monitoring
Source: Sensors (Basel). 2026 Jul 2;26(13):4177. doi: 10.3390/s26134177 (PMC13364356; doi:10.3390/s26134177)
Supplement: Supplementary file 1 [file sensors-26-04177-s001.zip › sensors-4335920-supplementary.pdf]

Figure S1 Pearson Correlation Analysis of Physiological Indicators

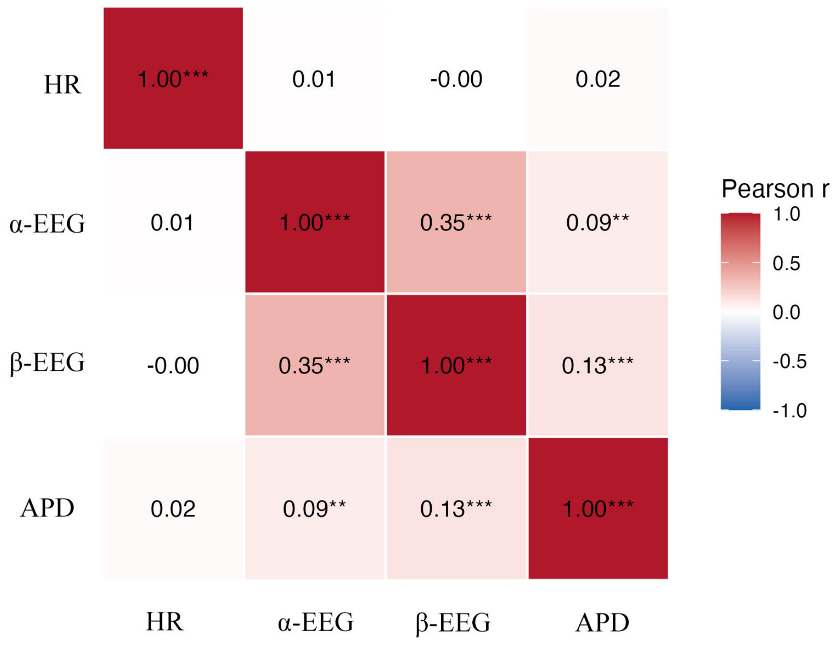

Table S1 Spatial visual and acoustic indicators

Types	Indicators	Description	Calculation method*
Visual	Openness (OP)	Percentage of prospect view pixels ($PVPixel$) of the street view image	$OP = \frac{PVPixel}{P}$
	Green view index (GVI)	percentage of greenery pixels ($GreeneryPixel$) of the street view image	$GVI = \frac{GreeneryPixel}{P}$
	Concealment (CM)	percentage of obstructed pixels ($ConcealmentPixel$) in the street view image	$CM = \frac{ConcealmentPixel}{P}$
	Material hardness ratio (MH)	ratio of the percentage of soft material pixels ($SoftPixel$) to the percentage of hard material pixels ($HardPixel$) in the street view image	$MH = \left(\frac{SoftPixel}{HardPixel}\right) \times 100\%$
	Infrastructure ratio (IC)	percentage of infrastructure coverage pixels ($InfrastructurePixel$) in the street view image	$IC = \frac{InfrastructurePixel}{P}$
	Elevation (EV)	The elevation value at this space	N.A.
	Historic buildings ratio (HB)	percentage of historic buildings coverage pixels ($HistoricbuildingPixel$) in the street view image	$HB = \frac{HistoricbuildingPixel}{P}$

	Colour richness (CR)	Number of colours in the street view image	N.A.
Acoustic	Natural sound (NS)	Average percentage of natural sound (T_{ns}) duration in the recorded segment	$NS = \frac{1}{n}\sum_{i=1}^{n}\frac{T_{ws,i}}{T_{total,i}}$
	Artificial sound (AS)	Average percentage of artificial sound (T_{as}) duration in the recorded segment	$AS = \frac{1}{n}\sum_{i=1}^{n}\frac{T_{ws,i}}{T_{total,i}}$
	Mechanic sound (MS)	Average percentage of mechanic sound duration (T_{ms}) in the recorded segment	$MS = \frac{1}{n}\sum_{i=1}^{n}\frac{T_{bs,i}}{T_{total,i}}$

*P is the total pixels of the street view image; T_{total} is the total number of recorded segments.

Table S2 Description of Physiological, EEG, and Eye-Tracking Indicators

Hierarchy	Indicators	Description
Psychological	Heart rate (HR)	Elevated activity and stressful states and its stimulation result in an increase in HR. In the quiet and relaxing states, HR decreases (Pham et al., 2021).
	Respiratory rate (RESP)	A high respiratory rate is typically associated with stress, anxiety, and tension. A low respiratory rate is usually associated with relaxation and meditative states (Pham et al., 2021).
	LF/HF	A higher LF/HF ratio suggests the dominance of sympathetic activity (stress or arousal), whereas a lower ratio indicates higher parasympathetic activity or relaxation (Immanue et al., 2023).
Electroencephalography	α-EGG (8-13Hz)	Increased alpha activity is commonly observed when an individual is in a relaxed, meditative state, or during quiet wakefulness with eyes closed (Laufs et al., 2003).
	β-EGG (13-30Hz)	High beta activity is associated with heightened cognitive activity, attention, stress, and anxiety (Palacios-García et al., 2021).
	β/α	A higher β/α ratio often indicates increased stress and anxiety levels, and is also associated with enhanced attention during focused cognitive tasks，while a lower ratio suggests a more relaxed state (Palacios-García et al., 2021).
Eye-Tracking	Average pupil diameter (APD)	An increase in pupil size (pupil dilation) is often associated with higher cognitive load, increased attention, and stress or arousal. Conversely, pupil constriction can indicate a state of relaxation or low cognitive demand (Beatty & Lucero-Wagoner, 2000).
	Fixation frequency (FF)	Higher fixation rates can indicate sustained attention and detailed processing of visual information, whereas lower fixation rates might suggest distraction or a broader, less focused scan of the environment (Holmqvist, 2011).
	Saccade frequency (SF)	A higher saccade rate can indicate exploratory behaviour and the search for new information and is often associated with increased cognitive load or stress. A lower saccade rate may indicate focused attention and fewer transitions between visual targets (Holmqvist, 2011).

Table S3 Kruskal – Wallis Test Results

Spatial Feature	Indicator	Chi-Square	p-value	Significance Level
Openness	HR	15.001	0.020	*
	α-EEG	28.444	0.000	**
	β-EEG	29.827	0.004	**
	APD	28.248	0.000	**
	Comfort	46.908	0.000	**
	Elevation	85.739	0.000	**
	Accessibility	73.355	0.000	**
	Social	31.590	0.000	**
	Tranquility	102.427	0.000	**
	Disorder	117.441	0.000	**
	Vibrant	260.531	0.000	**
	Uneventful	130.431	0.000	**
Natural Soundscape	HR	10.117	0.182	-
	α-EEG	23.782	0.001	**
	β-EEG	16.737	0.020	*
	APD	99.232	0.000	**
	Comfort	27.501	0.001	**
	Elevation	50.863	0.000	**
	Accessibility	38.589	0.000	**
	Social	21.998	0.002	**
	Tranquility	36.464	0.000	**
	Disorder	43.912	0.000	**
	Vibrant	176.305	0.000	**
	Uneventful	158.336	0.000	**
Natural Landscape	HR	10.004	0.185	-
	α-EEG	15.115	0.001	**
	β-EEG	13.070	0.004	**
	APD	27.919	0.000	**
	Comfort	29.865	0.000	**
	Elevation	66.814	0.000	**
	Accessibility	48.846	0.000	**
	Social	8.977	0.029	*
	Tranquility	79.776	0.000	**
	Disorder	95.218	0.000	**
	Vibrant	225.937	0.000	**
	Uneventful	114.257	0.000	**
Elemental Complexity	HR	18.437	0.002	**

α-EEG	15.655	0.007	**
β-EEG	10.429	0.064	-
APD	68.428	0.000	**
Comfort	34.155	0.000	**
Elevation	39.278	0.000	**
Accessibility	41.764	0.000	**
Social	20.642	0.009	**
Tranquility	26.113	0.000	**
Disorder	40.535	0.000	**
Vibrant	163.674	0.000	**
Uneventful	127.990	0.000	**

Table S4 Training set and test set results of random forest

Space Type	Physiological Indicator	Data Set	RESM	MAE	MBE	R^2
Path Platform	HR	Training Set	0.074	0.057	0.001	0.771
	HR	Test Set	0.139	0.113	-0.011	0.273
	α-EEG	Training Set	0.226	0.071	-0.004	0.566
	α-EEG	Test Set	0.364	0.139	0.012	0.062
	β-EEG	Training Set	0.054	0.038	-0.001	0.809
	β-EEG	Test Set	0.104	0.082	-0.019	0.300
	APD	Training Set	0.056	0.040	-0.001	0.870
	APD	Test Set	0.080	0.064	-0.003	0.649
Elevated Point	HR	Training Set	0.084	0.066	0.000	0.742
	HR	Test Set	0.153	0.114	0.032	0.088
	α-EEG	Training Set	0.034	0.026	0.000	0.823
	α-EEG	Test Set	0.069	0.053	0.001	0.322
	β-EEG	Training Set	0.051	0.036	0.000	0.801
	β-EEG	Test Set	0.087	0.064	0.003	0.419
	APD	Training Set	0.071	0.049	-0.001	0.798
	APD	Test Set	0.112	0.082	0.000	0.370
Viewing Boundary	HR	Training Set	0.072	0.052	-0.001	0.775
	HR	Test Set	0.133	0.108	0.000	0.205
	α-EEG	Training Set	0.044	0.032	-0.001	0.756
	α-EEG	Test Set	0.091	0.064	0.008	0.150
	β-EEG	Training Set	0.057	0.042	-0.001	0.791
	β-EEG	Test Set	0.086	0.066	-0.001	0.205
	APD	Training Set	0.048	0.035	0.000	0.883
	APD	Test Set	0.098	0.078	0.001	0.493
Key Node	HR	Training Set	0.069	0.050	0.000	0.768
	HR	Test Set	0.146	0.101	0.015	0.149

α-EEG	Training Set	0.040	0.030	-0.001	0.785
α-EEG	Test Set	0.059	0.047	-0.007	0.460
β-EEG	Training Set	0.054	0.038	-0.002	0.785
β-EEG	Test Set	0.078	0.062	-0.025	0.431
APD	Training Set	0.063	0.042	0.000	0.802
APD	Test Set	0.080	0.067	-0.011	0.613
